# HDL-Based Therapy: Vascular Protection at All Stages

**DOI:** 10.3390/biomedicines11030711

**Published:** 2023-02-27

**Authors:** Anastasia V. Poznyak, Vasily N. Sukhorukov, Ilya I. Eremin, Irina I. Nadelyaeva, Nikita A. Gutyrchik, Alexander N. Orekhov

**Affiliations:** 1 Institute for Atherosclerosis Research, Osennyaya 4-1-207, 121609 Moscow, Russia; 2 Institute of General Pathology and Pathophysiology, Russian Academy of Medical Sciences, 125315 Moscow, Russia; 3 Petrovsky National Research Centre of Surgery, 2, Abrikosovsky Lane, 119991 Moscow, Russia

**Keywords:** CVD, cardiovascular disease, atherosclerosis, ASCVD, HDL, cancer, diabetes mellitus

## Abstract

It is known that lipid metabolism disorders are involved in a wide range of pathologies. These pathologies include cardiovascular, metabolic, neurodegenerative diseases, and even cancer. All these diseases lead to serious health consequences, which makes it impossible to ignore them. Unfortunately, these diseases most often have a complex pathogenesis, which makes it difficult to study them and, in particular, diagnose and treat them. HDL is an important part of lipid metabolism, performing many functions under normal conditions. One of such functions is the maintaining of the reverse cholesterol transport. These functions are also implicated in pathology development. Thus, HDL contributes to vascular protection, which has been demonstrated in various conditions: Alzheimer’s disease, atherosclerosis, etc. Many studies have shown that serum levels of HDL cholesterol correlate negatively with CV risk. With these data, HDL-C is a promising therapeutic target. In this manuscript, we reviewed HDL-based therapeutic strategies that are currently being used or may be developed soon.

## 1. HDL Overview

High-density lipoprotein (HDL) is a small, dense lipoprotein with a mean size of 8–10 nm and density of 1.063–1.21 g/mL. HDL particles are rich in protein consisting of apolipoproteins, enzymes, and acute-phase proteins, and they may contain small amounts of nonpolar lipids. This variability of components leads to the high heterogeneity in structural, chemical, and biological properties. It was recently proposed to distinguish the followings subclasses of HDL: very large, large, medium, small, and very small HDL. This classification is based on the physical properties and unifies the data from several methods. HDLs are high-density lipoprotein particles that take part in reverse cholesterol transport from peripheral tissues back to the liver. HDL protection against CVD may be implemented via reverse cholesterol transport. HDL contributes to the cholesterol efflux from macrophages as well, which may proceed via passive diffusion or due to the action of the following transporters found on the walls of blood vessels [1]. ATP-binding cassette transporter G1 (ABCG1) facilitates the efflux of cholesterol into spherical large HDL, and ATP-binding cassette transporter A1 (ABCA1) uses apoA1 as an acceptor of lipids. Another variant of cholesterol efflux involves the SR-B1 (scavenger receptor B1). HDL has a special effect on atherosclerosis due to its pleiotropic actions, such as antioxidant and antithrombotic action, along with its ability to prevent a pro-inflammatory cytokine release, protect low-density lipoprotein (LDL) against oxidative modification, suppress cell adhesion molecule expression, stimulate nitric oxide generation, and inhibit aggregation of platelet molecules [2].

It has been demonstrated that serum levels of HDL cholesterol correlate negatively with CV risk. However, new studies in which the methods of raising HDL-C levels with CETP inhibitors and niacin were investigated did not reveal any decrease in cardiovascular risk [3,4,5]. Numerous cohort studies of new methods of HDL functionality assessment have shown that CV risk is much better determined by HDL functions, such as HDL cholesterol efflux capacity from macrophages, rather than by its concentration [6]. Diabetes is also linked to changes in both the structure and function of HDL. Recent studies revealed many factors contribute to HDL dysfunction in type 2 diabetes mellitus. Due to the glycation and oxidation of HDL-associated proteins, they become inactive. By changing the expression of genes and the activity of enzymes that metabolize HDL, metabolic disorders associated with diabetes reduce the efficiency of reverse cholesterol transport. Finally, an ever-increasing inflammatory state leads to major changes in the HDL proteome; changes that not only deprive HDL of its normal function but also turn it into a pro-atherogenic particle [7].

In this review, we summarized the data on HDL, focusing on the three important and widespread pathologies, Alzheimer’s disease, atherosclerosis, and diabetes mellitus. We selected papers to review from the PubMed and Google Scholar databases. We mostly obtained papers published in the past five years, including older ones when it was necessary. To find initial publications, we used keywords, such as “HDL”, “HDL in atherosclerosis”, “HDL in Alzheimer’s disease”, etc.

## 2. HDL-C Levels vs. HDL Function

HDL-C levels in plasma negatively correlate with the risk of atherosclerosis, as was demonstrated at all LDL-C levels [8]. HDL takes part in RCT as a lipid acceptor, which stimulates cholesterol efflux from macrophage foam cells and transports the cholesterol to the liver and bile. Attempts to raise HDL levels with niacin resulted in a 30% elevation of HDL-C concentration but failed to improve its function [9]. While CETP inhibitors and niacin raised HDL-C levels in serum significantly, in randomized controlled trials it did not reduce the cardiovascular risk [10]. Mendelian randomization studies of variants associated with elevated HDL-C levels (e.g., P376L) showed no connection between those variants and CV risk reduction. In animal models, a decrease in cardiovascular risk is generally influenced by cholesterol efflux capacity (CEC) and not HDL-C levels in serum. The research of the measure of CEC and HDL function in low-HDL and high-HDL serum samples (HDL ≤ 10th and HDL ≥ 90th age/sex-specific percentile) showed an impaired capacity of cholesterol acceptors in serum obtained from specimens with low HDL levels [11]. While HDL-C concentration measurement might give information on the HDL particle number, its function and structure are not evaluated correctly, so there is a need to develop standardized tests to assess HDL functionality for clinical use. A high-throughput cell-free test was developed as an HDL function surrogate based on lipid peroxidation of HDL, and it was reported to be consistent with CV events risk [12]. The Dallas Heart Study [13] and other studies with big groups of people evaluated CEC as a measure of HDL function using cell techniques, such as fluorescent reagents (e.g., bodipy—boron dipyrromethene difluoride) and radioactively labeled cholesterol. The highest quartile indicated a 67% decrease in cardiovascular risk compared with the lowest quartile of CEC. Thereby, CVD risk may be predicted by CEC along with other factors. Controlled trial studies indicated that the infection of HIV is related to a decrease in HDL-C levels and, thus, elevated cardiovascular risk. CEC has been assessed in individuals with acute HIV who were randomized to receive antiretroviral therapy (ART). After the ART treatment for the patients with HIV and a reduction in viral load, the expression of ABCA1 was increased, which induced CEC elevation. The EPIC-Norfolk study of CEC evaluation indicated that cholesterol efflux is an independent and accurate measure of the HDL atheroprotective potential [14].

## 3. HDL in Pathology

In this section, we summarized the potential and currently known roles of HDL-based therapy strategies for the management of various diseases. We provide the most significant data in Table 1 and Table 2.

### 3.1. Considerations to Evaluate HDL as a Potential Therapeutic Target for the Vascular Contribution to Alzheimer’s Disease

Treatment approaches to protect or recover the blood–brain barrier (BBB) based on HDL are endorsed by several studies in vitro and in both animals and humans. Several treatment modalities for cardiovascular disease have been clinically tested and have data on safety and effectiveness. ApoA1 mimetics (e.g., L-4F and D-4F), recombinant apoA1 protein CER-001, a formulation of apoA1 CSL-112 obtained from plasma, and autologous administration of apoA1 obtained from the patient all showed good tolerability results in the first phase of clinical trials for ACS or stable coronary heart disease [29]. Development of some of these substances was stopped after they failed to demonstrate any positive impact on HDL function or a decrease in atherosclerosis. However, autologous apoA1 and CSL-112 treatments have displayed potential and will go through phase III clinical trials (NCT03135184, NCT03473223) [30].

Indirect therapeutic agents based on HDL comprise CETP inhibitors, niacin, lecithin–cholesterol acyltransferase recombinant protein ACP-501, and apoA1 transcription up-regulator RVX-208. RVX-208 did not show an atheroprotective effect and led to a dose-dependent elevation of liver transaminases [31]. ACP-501 demonstrated good tolerability results in patients with stable coronary disease and is going through a phase II clinical trial designed to evaluate its impact on apoB metabolism in individuals with coronary heart disease (NCT03773172) [32]. Niacin therapy was assumed to lower CVD and atherosclerosis risk in early studies. However, two randomized controlled trials were later halted due to no apparent positive effects. Some CETP inhibitor studies were stopped prematurely due to inefficacy and safety issues, such as elevated mortality with torcetrapib [33]. Anacetrapib, which is undergoing phase III clinical trials, did not show any negative effects and decreased the risk of CV events. Some CETP polymorphisms are connected to Alzheimer’s disease development and memory impairment, especially in patients with apoE4. Therefore, CETP inhibitors can be particularly useful in Alzheimer’s disease treatment [34].

### 3.2. Evaluation of HDL-Based Therapeutics on Alzheimer’s Disease-Relevant Outcomes in Animal Models

Therapeutic approaches based on HDL have not been studied for Alzheimer’s disease in clinical trials. However, several preclinical trials have been conducted in an Alzheimer’s disease murine model. Reconstituted HDL administration caused a decrease in the concentration of soluble brain antibodies in mice with APP/PS1 and with SAMP8 [15]. Memory deficiency and microgliosis were lowered as well. Intravenous recombinant apoA1 Milano administration decreased microgliosis, cerebral amyloid angiopathy (CAA), and deposition of antibodies in mice with APP23. In mice with APPswe/PS1DE9, oral D-4F therapy led to the amelioration of antibody deposition, memory, astrogliosis, microgliosis, and other inflammation markers. Aside from Alzheimer’s disease therapeutic approaches, D-4F therapy following the occlusion of a middle cerebral artery caused a decrease in neuroinflammation and injury of white matter. In mice with atherosclerosis, it also ameliorated cognition and lowered inflammation of the cerebral arterioles [16].

### 3.3. Additional Lipid-Modifying Therapeutics for the Prevention and Treatment of Dementia

Another method of Alzheimer’s disease treatment could be lipid-modifying therapy that does not target HDL directly. Statins stop the synthesis of cholesterol and slightly raise the HDL-to-LDL ratio by inhibiting 3-hydroxy-3-methyl-glutaryl-coenzyme A (HMG-CoA) reductase. Meta-analyses show that the use of statins reduces the risk of dementia in prospective studies, although not in two randomized controlled trials [35]. Retrospective group studies demonstrated that niacin could ameliorate cognitive function 25 years after in cases of higher consumption at a young age. Alzheimer’s disease risk was also decreased, and cognitive function improved over six years of follow-up care in older patients with higher consumption. Unfortunately, the said studies did not evaluate niacin concentrations in the blood [36].

The HDL biogenesis rate-limiting stage includes efflux mediated by ABCA1. Therefore, other possible drugs for indirect HDL treatment might be compounds that target ABCA1, e.g., retinoid-x-receptor (RXR) and liver-x-receptor (LXR) agonists. Direct RXR and LXR agonists raise CNS apoE lipidation and HDL cholesterol concentrations in plasma and improve cognition in animal models of Alzheimer’s disease [37]. Substantial systemic and hepatic adverse effects hinder further RXR and LXR development. However, novel ABCA1 modulators independent of LXR could be the solution to this issue. Bexarotene, the RXR agonist, was the first drug targeting ABCA1 that was used in clinical studies. In a phase I trial, it increased cerebrospinal fluid apoE concentration, but the bioavailability was low [20].

### 3.4. Efficacy of HDL as a Potential Therapeutic Target to Protect and Repair the Cerebrovasculature in Alzheimer’s Disease

Multiple therapeutic agents based on HDL have significant proof of their safety, which confirms that those agents might be possibly redirected for the treatment of AD. According to the positive impact of HDL in human arteries bioengineered in 3D and murine models, HDL could be useful for the prevention of CAA and neuroinflammation associated with Alzheimer’s disease. Moreover, HDL could be used to carry microRNAs and drugs and, thus, solve the problem of penetrance of the BBB. In mice with Alzheimer’s disease, a reconstituted HDL proved to be able to get into the brain, ameliorate memory function and decrease amyloidosis while carrying an antibody-targeting drug [38].

### 3.5. HDL as a Potential Target for Treatment of Breast Cancer

It is possible to prepare HDL nanoparticles to carry apoA1 and phospholipids. Those particles have particular characteristics that could help deliver drugs. If these particles are produced in sizes smaller than 30 nm, they might enter cells, stay in the bloodstream for an extended time, and promote stabilization of hydrophobic substances in the core of HDL. Treatments that include HDL-mimetic nanoparticle intake show the ability to elevate HDL concentrations by 30 times without harm to cells (could be tolerated by cells) [39]. The nanoparticles can perform their functions auspiciously for the delivery of drugs by binding to scavenger receptor B1. HDL comprises the cholesterol esters that may be transported to the liver cells via the SR-B1. Thereby, the transferred substance is prevented from being degraded in lysosomes. HDL appears to be a good potential transporter of anticancer drugs to the target cells due to its high affinity and capability to build up in cancer cells [40]. Moreover, experimental data have demonstrated that the physical characteristics of drugs stored inside the HDL may be maintained, which has been confirmed by electron microscopy and high-performance liquid chromatography. Consequently, HDL properties and size were not altered by placing drugs inside the HDL. Anticancer medicinal compounds based on HDL may also help lower the adverse effects of chemotherapy. The release of chemotherapy drugs into cells necessarily requires reconstituted HDL involvement [41]. The rHDLs could be preferable for anticancer drug delivery because of their appropriate size and cancer cells’ affinity to absorption of the main HDL components. Quickly dividing tumor cells need cholesterol to form membranes. Absorption of the main components of HDL mediated by receptors could satisfy this need for additional cholesterol [42]. As was demonstrated in several trials, the proliferation of cells is related to HDL absorption and elevated HDL receptor expression in breast tumor cells. Generally, the results of these studies show that the beneficial properties of HDL allow these particles to act as drug transporters. Toxic adverse effects of anticancer medicines may be decreased by lowering HDL receptor expression in the peripheral cells as a result of the delivery of an HDL complex [43].

While the scavenger receptor B1 is excessively expressed, it is assumed that it might act as a cancer marker and a drug transporter. Multiple recent studies have shown that tumor development is related to SR-B1 expression [44]. Some evidence suggests that delivery of cholesterol to rapidly growing malignant cells, facilitated by overexpression of the SR-B1 receptor, is required in at least some tumor types [45].

Hormonal anticancer therapeutic approaches have an adverse effect on the lipid profile, e.g., in individuals with malignant neoplasms, the triglyceride concentration was increased, and the HDL cholesterol level was reduced. The significance of this influence is yet to be investigated. Pitavastatin therapy led to a rise in HDL cholesterol and the HDL antioxidative effect, improvement of the cholesterol delivery to the cells, and a decrease in LDL levels [46]. Therefore, pitavastatin increases HDL concentration, enhancing its capacity and preserving its quality. Several studies revealed a connection between breast tumors and oxidative stress. HDL might be subject to oxidative alterations in the case of oxidative stress, and thereby its function might be influenced by these changes [47].

### 3.6. HDL-Modifying Treatment Approaches in Diabetes

At present, diabetic dyslipidemia is mostly treated with statins that inhibit HMG-CoA reductase [48], which is a rate-controlling enzyme that takes part in the biosynthesis of cholesterol, leading to HDL and apoA1 elevation, and a decrease in LDL and TGs. In vitro studies of pitavastatin demonstrated its ability to raise cholesterol efflux and improve HDL antioxidative action by raising PON-1 [49]. There are several potential explanations for such effects, all of which are based on various mechanisms. Previously, it was shown that pitavastatin increases apoA1 production, as well as the production of ABCA1 in vitro. In addition, a decrease in endothelial lipase expression, which increases the amount and particle size of HDL, was observed. These phospholipid-rich HDL particles exhibited antioxidant and anti-inflammatory effects. Considering that cholesterol efflux is crucial for this process, ABCA1 and ABCG1 appeared to be upregulated. To sum up, pitavastatin seems to act via a dual mechanism increasing HDL numbers, thus inhibiting HDL catabolism and stimulating HDL synthesis. However, these results contradict other studies that indicate a decrease in cholesterol efflux mediated by ABCA1 after statin treatment [50]. Nonetheless, a meta-analysis of statin treatment demonstrated a substantial decrease in mortality and CV events, including stroke and myocardial infarction, in more than 18,000 individuals with type 2 diabetes mellitus. Studies of individuals with type 1 diabetes mellitus showed analogous results. However, the study of Voight et al. showed the potential existence of genetic mechanisms that raise plasma HDL cholesterol, an action which does not seem to lower the risk of myocardial infarction. This challenges the hypothesis that raising plasma HDL cholesterol will uniformly translate into reductions in the risk of myocardial infarction [51]. The latest studies of high-intensity statin treatments demonstrated regression of atherosclerotic plaques in patients with and without diabetes [21]. Although, there might be certain issues related to the ability of statins to elevate T2DM risk, particularly in individuals with metabolic syndrome and other conditions. Numerous meta-analyses support these data and indicate an elevation of incident DM risk as such an effect of statins. The reasons for this effect are yet to be discovered, although, there was some negative impact involved in insulin sensitivity and function of pancreatic β-cells and gene polymorphisms in HMG-CoA. Nevertheless, the decrease in CV risk appears to be more essential compared with the mild increase in incident diabetes risk [52].

There were several studies conducted on an inhibitor of intestinal absorption of cholesterol via the NPC1L1, ezetimibe. It was inspected as an adjunct to statin treatment, particularly in patients with insufficient control of LDL concentration. Combined use of statins and ezetimibe showed a more considerable decrease in LDL levels and CVD risk in individuals with diabetes in comparison to the regular or double-dose use of statins alone. The impact on HDL levels is still debatable due to the contradictory results of different studies. Nonetheless, the recent study IMPROVE-IT demonstrated that the combined use of ezetimibe and simvastatin lowered CVD risk notably in individuals after ACS [22]. Additional subgroup analysis revealed that this positive effect was more significant in those with diabetes mellitus. In a T2DM murine model, ezetimibe therapy was proven to raise the number of pancreatic β-cells as well as enhance the effect of glucagon-like peptide-1, an incretin hormone that promotes the release of insulin, shedding light on some benefits of ezetimibe [19].

In the context of therapy for diabetic dyslipidemia, some interest has recently been generated by monoclonal antibodies, which inhibit proprotein convertase subtilisin/kexin type 9 (PCSK9) attachment to receptors of LDL, thus protecting them from destruction in the lysosomes. Evolocumab and alirocumab are presently available inhibitors of PCSK9; they are able to lower LDL levels more effectively compared with statins, normally by 50–60%, and slightly increase HDL levels by 7–11% [53]. These impacts seem to be the same in individuals with or without type 2 diabetes. Evolocumab first demonstrated an enhancement of CV events in individuals with ASCVD receiving statin therapy during the FOURIER trial [54]. The subgroup analysis revealed a similar decrease in CV risk in individuals with and without diabetes. Alirocumab showed an ability to decrease LDL as well in new randomized studies, which included individuals with type 1 and 2 diabetes receiving maximal doses of statins. In comparison to regular lipid-lowering treatment in individuals with type 2 diabetes, this effect was also significant. Notwithstanding that, Mendelian randomized trials indicated a connection between gene variants of PCSK9 related to reduced LDL and a higher risk of developing diabetes. Luckily, this correlation was not confirmed in clinical studies. PCSK9 inhibitors could have big potential as therapeutics due to their safety and efficacy [55].

Some other HDL-increasing drugs were studied lately in individuals with diabetes but did not show any promising outcomes. Extended-release niacin therapy resulted in a decrease in LDL and TGs and an increase in HDL concentration by 35%. In individuals with type 2 diabetes, it improved the attenuated ability of the HDL to fight oxidative stress, repair endothelium through EPCs, and enhance endothelial nitric oxide (NO) formation. However, two large clinical trials, HPS2-THRIVE and AIM-HIGH, did not indicate any considerable benefit from niacin treatment applied in addition to statins in individuals with and without diabetes. Niacin treatment did not show any influence on HDL anti-inflammatory features or on CEC, which could be connected to the interference of statins, whereas it did increase the levels of HDL. Furthermore, niacin clinical use is limited due to its adverse effect of flushing and a higher risk of developing diabetes [23].

CETP inhibitors such as dalcetrapib, torcetrapib, and evacetrapib showed no considerable positive effects either on CV risk in individuals with or without diabetes, even though they did increase the HDL concentrations. On the contrary, torcetrapib treatment elevated CV risk and mortality rate [24]. The new REVEAL trial of anacetrapib was the first to demonstrate a decrease in coronary artery disease events associated with increased HDL concentration and reduced non-HDL cholesterol concentration in individuals receiving statin therapy [25]. The risk of developing diabetes was decreased as well compared with the patients in the placebo groups. In fact, it was revealed earlier that HDL obtained from individuals receiving anacetrapib increases cholesterol efflux from macrophages and retains anti-inflammatory characteristics through ABCG1 and ABCA1-dependent pathways. Nevertheless, there is an assumption that the positive impact of anacetrapib was a result of the non-HDL cholesterol decrease and not the HDL increase [56].

Considering some effects of these agents, there is growing interest in the engineering of analogs of HDL and apoA1 mimetic peptides for CVD therapy. Research on these new drugs has been mostly limited to animal models and in vitro studies, but some of them demonstrate potential. For example, D-4F therapy led to a substantial decrease in the infiltration of macrophages and atherosclerotic lesions in a diabetic atherosclerosis mouse model [57].

In the past few years, increased attention has been paid to the microRNA role in the pathophysiology of CV events in patients with diabetes mellitus. MicroRNAs are composed of non-coding single-stranded short (21 to 24 nucleotides) molecules of RNA. These molecules post-transcriptionally regulate the mRNA of the target gene via specific base pairing with their 3’ UTR, thus suppressing translation and contributing to their destruction [58]. The impact of multiple microRNAs on diverse types of cells and their functionality makes them associated with adverse CV events in patients with diabetes and a potential marker or therapeutic target. Data indicate that HDL and other LPs may in fact deliver microRNAs stored within the target cells. MicroRNAs may modulate HDL metabolism and functionality, e.g., miR-33a and miR-33b, which presumably take part in the synthesis of HDL and cholesterol efflux. It is still unclear if the HDL impact in DM and its CV event are mediated by microRNAs [59].

### 3.7. HDL as a Target for Atherosclerosis Treatment

It is widely known that following a healthy lifestyle and eating a healthy diet is associated with a reduced risk of developing cardiovascular disease. Therefore, it is quite logical that their relationship with HDL has also been studied, including in humans. In these studies, in people at risk for developing cardiovascular disease, HDL-C levels as well as HDL functionality were shown to improve with MUFAs, PUFAs (particularly long-chain, omega-3 MUFAs and PUFAs in fish), and dietary antioxidants, such as phenolic compounds (in dietary or near-dietary doses). Based on this, an antioxidant-rich diet resulted in improved HDL function in both healthy individuals and those at high cardiovascular risk. It is important to note that the increase in HDL levels is higher in individuals with initially higher levels. This contributes to the significant but not sufficient role of healthy lifestyle, exercise, and nutrition [60].

**Figure 1:**
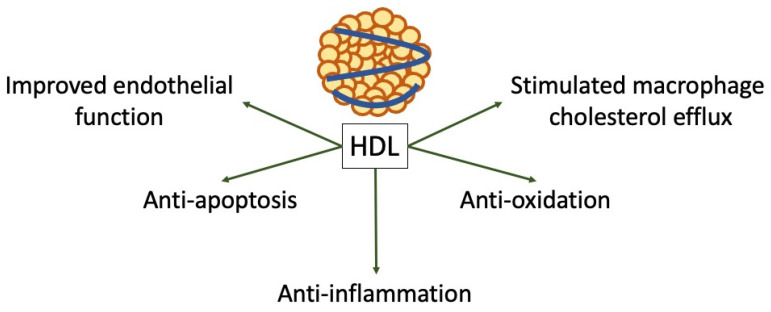
Anti-atherosclerotic effects of HDL.

In Figure 1, we summarized the range of anti-atherosclerotic effects of HDL.

The early studies of the HDL role in atherosclerosis were carried out in animal models, e.g., rabbits that were fed with cholesterol and then injected with HDL to lessen the lesions. The lesion development was reduced, and the established lesions showed regression after infusion of homologous HDL. Later, those researchers reported multiple features of this atheroprotective action, such as a decrease in the release of prostacyclin in SMC depending on the expression of cyclooxygenase-2 [61].

The invention of the intravascular ultrasound (IVUS) examination was a medical advancement. A trial of IVUS and standard coronary angiography demonstrated a negative association of HDL cholesterol concentrations with a thickness of plaque, defined as the maximum intima index, in individuals with hereditary combined hyperlipidemia. These results drew attention to a need for a trial with the mutant apolipoprotein A1 Milano as a potential atheroma regression stimulator. There was a significant decrease in HDL cholesterol concentrations in patients with the mutant (Cys-Arg substitution at position 173 of apoA1) in the case of strong cardioprotection. Trials of the turnover showed that the apoA1 Milano dimer (A1M/A1M) exhibits a superior ability to transfer cholesterol from tissues and good blood stability after one injection (10 days versus 5 days for the protein of wild type) [26].

The dimeric apoA1 Milano was tested afterward to assess its influence on a focal atheroma. The latter was induced by electrocution of the CCAs of rabbits on a high-cholesterol diet and was associated with a major cumulation of lipids and macrophages. Direct evaluation of local apoA1 Milano delivery was performed through the ECA, placing the catheter tip proximal to the focal atheroma [62]. ApoA1M–DPPC compounds were administered as single doses of protein (0.25–1 g) within 1.5 h. As a result, the plaques had shrunken significantly by up to 30%. A histochemical analysis verified the direct impact on lipid disposal. The long-term persistence in the atheroma of the immunochemically detectable apoA1M was also established during the evaluation following animal euthanasia after three days. Another study conducted by Spanish researchers indicated apoA1M presence in the aortas six months after two doses of the dimeric apoA1M [63].

The achievements of these animal model trials led to the start of clinical assessment with analogous protocol in acute coronary syndrome individuals after invasive treatment. The participants with 20 and higher percent narrowing of the lumen of coronary arteries were randomly divided to be treated with five injections of 15 or 45 mg/kg dose [64]. There was a 4.2% reduction in the overall volume of the plaques from the original level in the therapy groups, whereas the placebo patients showed none. The individuals with the highest original volume of plaques demonstrated the greatest effect [65].

More thorough research of said effect was possible after the developer (Esperion Therapeutics) was taken over by Pfizer. Sadly, a new apoA1 Milano drug treatment caused serious allergic reactions in ACS patients and one lethal outcome. Those consequences were caused by small protein impurities in the new compound. The clinical studies were terminated afterward notwithstanding the fundamental research with alternative treatment modalities in progress. Presently the Medicines Company (MDCO) is in charge of the apoA1 Milano development [66].

In the past few years, there have been tests performed, primarily with the IVUS, of three agents: the dimeric apoA1 Milano and two normal apoA1 preparations combined in diverse compositions. Various constituents of PL were also assessed in reconstituted (r) products. The most chosen PL constituents in rHDL in animal and clinical studies were 1-palmitoyl-2-oleylphosphocholine (POPC) and dimyristoylphosphatidylcholine (DMPC), and recently, the added tests of Sph and PL were also conducted. POPC was found to be simpler in use, as it is fairly stable, not too expensive, and has mild fluidity; hence, it is primarily selected for clinical trials [67]. Newly reported anti-inflammatory characteristics of SM as a PL constituent and the HDL lipid enrichment with PS proved to exert more beneficial features in vitro and murine models compared with the particles without PS [68].

The rHDL (CSL-111) obtained from blood was administered in high doses (40 or 80 mg/kg) four times over four weeks, which led to a modest but not considerable decrease in the volume of atheroma in comparison to the placebo group; however, a substantial coronary score alteration was indicated (ERASE trial) [69]. The trial of the 80 mg/kg dose was terminated early due to liver adverse effects. That novel drug accessibility presented an opportunity to assess the injections’ direct impact on individuals with atherosclerosis of the common femoral artery (CFA). The rHDL dose administered once led to a decrease in the size of macrophages, mediators of inflammation (e.g., VCAM-1), and plaque lipids concentration. The purified product (CSL-112) was reported in the AEGIS-1 study to be safe and effective in stimulating cholesterol efflux mediated by ABCA1; however, it was not connected to the regression of atheroma by IVUS.

As CSL-112 demonstrated an ability to induce cholesterol efflux from foam cells equally in patients with and without stable atherosclerosis, it thus became a subject of assessment in a randomized controlled study of a single 6 g apoA1 injection in 17 thousand individuals with MI (AEGIS II trial). Although, due to the small dosage, the apoA1 administration might not have been enough to diminish the ischemic damage seven days after the infarction [70].

An analogous protocol was used to assess another HDL drug enriched with sphingosine from Cerenis (CER-001). Individuals with acute coronary syndrome were receiving six injections of CER-001 over six weeks: 3, 6, or 12 mg/kg or a placebo (CHI-SQUARE study). None of the doses led to a decrease in coronary atherosclerosis on QCA or IVUS. In a subsequent test, the infusion of the lowest dosage appeared to be efficient, although the next clinical study failed to confirm that result [27].

The MDCO-216 (apoA1 Milano and 1-palmitoyl-2-oleoyl-sn-glycero-3-phosphocholine) was tested by the same scientists again in 120 individuals, who received 20 mg/kg of the preparation (*n* = 52) or a placebo (*n* = 60) once a week for five weeks. This study also did not indicate any considerable atherosclerosis decrease. It is stated in an accompanying Editorial that the results of apoA1 Milano administration in stable and acute atherosclerosis might vary, showing positive results in the former case [28]. The authors also indicated that in one test in mice transfected with the apoA1 Milano gene, the cholesterol was not mobilized sufficiently, while in mice of wild type, this happened to a greater degree, thus inferring that apoA1 Milano may be of lower benefit in the context of direct CEC. There was a difference reported between macrophages obtained from animals with transfected genes and animals of wild type with the latter showing a greater ability to mobilize cholesterol. As an assessment of apoA1 Milano versus apoA1 on CEC in a gene-transfected murine model is disparate, Radar’s interpretation is not entirely substantiated [71]. Another group of researchers stated that the unaltered LP profile in individuals receiving apoA1 Milano is different from the LP profile in the previous trial by Nissen and colleagues. Moreover, in the last trial, apoA1 Milano increased hsCRP, which most likely means that several characteristics of the original apoLP structure might have been altered [72].

Hovingh and colleagues used another technology, MRI, to assess the HDL treatment effect, and they analyzed the CER-001 by 3-TMRI carotid scan [73]. Twenty-three individuals were confirmed to be homozygous or compound heterozygous for apoB, LDL-R, LDL RAP1, or PCSK9 and received 8 mg/kg of the drug twice biweekly for 12 weeks. As a result, apoA1 was elevated from an average of 114.8 ± 20.7 mg/dL to 129.3 ± 23 mg/dL. The average vascular wall area was diminished from 17.23 to 16.75 mm^2^ (*p* = 0.008). This trial demonstrated that HDL therapy leads to a decrease in vascular wall area, suggesting a decrease in plaque prevalence. In particular, the average thickness of the carotid artery wall was decreased by approximately 2.5% [74].

New scientific fields were researched to use the apoA1 Milano characteristics for the prevention and treatment of CVDs, one of them being the research of vascular gene therapy. Viral vector gene therapy has been auspiciously performed with the apoA1 in animals with apoE-KO. Wang and colleagues were the first to study the assumption that apoA1 Milano gene therapy may appear more efficient in atherosclerosis treatment than the standard administration of recombinant apoA1 Milano [75]. The test was performed by transplant of bone marrow in a female murine model of mice with absent apoE and apoA1. Bone marrow was obtained from mice with transduction by retroviral vector expressing WT apoA1 or apoA1 Milano. The donor bone marrow was then transplanted into double knockout (DKO) cholesterol-fed female mice, which were euthanized 24 weeks following the experiment. The atherosclerosis of the aorta was decreased by 25% with the apoA1 gene of wild type, which is 65% lower than the effect of apoA1 Milano. It is noteworthy that the circulating cholesterol concentrations were the same in both groups [76].

Recently, that group of researchers examined classical tactics of gene therapy and studied the recombinant adeno-associated virus 8 (rAAV-8) vector for apoA1 Milano in the apoE/apoA1 DKO murine model. The mice were put on a diet low in cholesterol after twenty weeks of high cholesterol one, and then they received an infusion of the rAAV8 vector either expressing apoA1 Milano or empty. After 40 weeks, the mice treated with rAAV8 apoA1 Milano demonstrated a substantial reduction of atherosclerosis, while there was no such positive effect in mice treated with empty rAAV8, and they were sacrificed before the experiment had started. It can be concluded that rAAV8 apoA1 Milano gene therapy exerts regression of atherosclerosis, while a low-cholesterol diet is only able to stop the atherosclerosis development but not stimulate its regression [77].

One more method of gene therapy includes the transducing of arterial endothelial cells with the HdAd vector expressing apoA1. During the experiment, rabbits were put on a high-fat diet and underwent two-sided carotid gene transfer. One artery received a vector expressing apoA1, and the other received an empty vector Hd Null. Results after 24 weeks showed that the volume of damage to the intima-media was 30% lower (*p* = 0.03), the content of muscle cells decreased by 32%, and the content of intimal macrophages decreased by 23% in rabbits treated with the apoA1-expressing vector [78]. ICAM-1, MCP-1, VCAM-I, and TNFα markers of intimal inflammation were reduced as well in arteries that received the apoA1-expressing vector. Therefore, vascular gene transfer could be very helpful in lowering inflammation of the intima and the growth of atherosclerosis lesions. Recently, this group of researchers indicated that a decrease in inflammation and elevation of CEC may be induced by simultaneous overexpression of ABCA1 [79].

A completely new method is to use the intestinal transport system to administer apoA1 Milano orally. Romano and colleagues studied this method on genetically engineered rice plants, using electroporation to put altered plasmids into Agrobacterium tumefaciens, which transformed Oriza sativa SSP Japonica Rosa Marchetti [80]. The gene modification was completed effectually, as indicated by the whole genome DNA derived from the rice plant leaves. Expression was detected in the rice seeds and pulps; the former was afterward converted to “rice milk”. Expression was insignificant in stems, roots, and leaves. The genetically modified “rice milk” special properties are listed in a patent (n° PCT/IB2006/054948). Curiously enough, the transfected apoA1 Milano was mostly found as a dimer, which was proved to be able to induce AS regression.

There were in vitro studies conducted of the transgenic “rice milk” on oxLDL-exposed THP1 macrophages to assess its action against inflammation. The transgenic “rice milk” was able to lower the formation of foam cells and inhibit the generation of MCP1 in proportion to the apoA1 Milano levels. Moreover, cholesterol efflux was elevated notably after administration of 0.1 or 0.5 μg/mL of apoA1 Milano. In summary, the “rice milk” caused no significant side effects in the apoE-/- murine model, providing an opportunity to assess its effect. The mice were getting the Western type of food for eight weeks and, in the next three weeks of the experiment, stayed on that diet. They received the “rice milk” via gastrogavage at a dose of 10 mL/kg five days a week [81].

The volume of atherosclerosis lesions was decreased substantially by approximately 50% as a result of the apoA1 Milano “rice milk” therapy; in mice fed with natural rice milk, there was no such effect. Considerable amelioration was detected at the level of the aortic arch and the level of the aortic sinus. These conclusions are still questionable. When administered through the oral route, apoA1 mimetic peptides are poorly uptaken by the small intestine. An improvement of the absorption might not be due to the ABCA1 or other HDL cell transporters’ involvement. The ability to treat patients with transgenic rice milk could prove very useful. Thus, these presumptive mechanisms have to be assessed in suitable animal or human models, as the intestine plays an important role in HDL biogenesis [82].

HDL treatment might be a useful method of lowering atherosclerosis in animals. Although some of the beneficial discoveries were not completely confirmed, it is still yet to be investigated why that happened. Moreover, lately, more distinctions have been detected in murine models of early- and advanced-stage atheroma [83]. The results of human apoA1 injection in apoE-/- cholesterol-fed mice with early-stage atherosclerosis differed from those of mice with an advanced stage. In the case of late-stage atherosclerosis, the administration of apoA1 caused a very modest impact on the volume of atherosclerosis lesions and on plaque structure. The mice with an early stage showed a notable regression of atherosclerosis: SMC content was raised, macrophage levels decreased by 51.2%, and plaque volume decreased by 30.2%. The researchers stated that the reasons might be attenuated CEC of plasma depleted of apoB and a decreased level of protection against inflammation and apoptosis in the case of the advanced-stage atherosclerosis. Thereby, anti-atherosclerotic and cellular actions of HDL therapy seem to be attenuated in the case of advanced-stage atherosclerosis, and that knowledge might be of help in forthcoming research [84].

## 4. Conclusions

HDL is known for its vasculoprotective properties. In our review, we focused on the beneficial properties of HDL in serious diseases closely related to the vascular system and metabolic disorders.

Some CETP polymorphisms are connected to Alzheimer’s disease development and memory impairment, especially in patients with apoE4. Therefore, CETP inhibitors can be particularly useful in Alzheimer’s disease treatment. Statins stop the synthesis of cholesterol and slightly raise the HDL-to-LDL ratio by inhibiting 3-hydroxy-3-methyl-glutaryl-coenzyme A (HMG-CoA) reductase. Diabetic dyslipidemia is also mostly treated with statins which inhibit HMG-CoA reductase.

Anticancer medicinal compounds based on HDL may also help lower the adverse effects of chemotherapy. The release of chemotherapy drugs into cells necessarily requires reconstituted HDL involvement.

HDL treatment may affect a large variety of targets in clinical atherosclerosis and related areas. The isolated HDL demonstrated its positive effects back in the 1990s, and those results have been replicated numerous times. Various indirect treatment options can be a good variant to lower CVD risks in different subjects as well as having the potential to lower the atherosclerosis rate. However, these therapeutic strategies still need to be investigated further.

## Figures and Tables

**Table 1:** Summary of animal studies targeting HDL in various pathologies.

Drug	Dosing	Effects on HDL Level	General Effects	Subjects	Reference
Reconstituted HDL administration	injected i.v. via the tail vein once weekly for 4 weeks with 60 mg/kg rHDL	not tested	decrease in concentration of soluble brain antibodies; decrease in memory deficiency and microgliosis	mice with APP/PS1 and with SAMP8	[15]
Intravenous recombinant apoA1 Milano administration	2 mg/kg of rApoA1M	no significant differences from the control group	decreased microgliosis, cerebral amyloid angiopathy (CAA) and deposition of antibodies	mice with APP23	[16]
oral D-4F therapy	200 µg/mL with water	not tested	amelioration of antibody deposition, memory, astrogliosis, microgliosis, and other inflammation markers	mice with APPswe/PS1DE9	[17]
oral D-4F therapy	500 µg (dissolved in 200 μL of water and given by stomach tube)	significant decrease in HDL lipid hydroperoxides 20 min after oral D-4F	also ameliorated cognition and lowered inflammation of the cerebral arterioles	ApoE-null mice	[18]
Ezetimibe	10 mg/kg for 6 weeks	not tested	increased number of pancreatic β-cells; enhanced effect of glucagon-like peptide-1	male db/db mice aged 8 weeks	[19]

**Table 2:** Summary of clinical studies targeting HDL in various pathologies.

Drug	Dosing	Effect	Study Details	Subjects	Reference
Bexarotene	300 mg of bexarotene for 4 weeks	elevated triglycerides (potential increase in CV risk)	double-blind, placebo-controlled, proof-of-concept trial	20 Alzheimer’s disease patients with positive florbetapir scans	[20]
Statins	various schemes	21% reduction in major vascular events per mmol/L LDL-C reduction	meta-analysis of 14 trials	18,000 diabetes mellitus patients	[21]
Ezetimibe + statins	adding ezetimibe 10 mg/day versus placebo to ongoing, open-label statins treatment for 8 weeks	lowered CVD risk	post hoc analysis of data from a randomized, double-blind, placebo-controlled trial	769 hypercholesterolemic patients	[22]
Niacin	various schemes	controversial results	meta-analysis of randomized controlled trials	2110 diabetes mellitus patients	[23]
Torcetrapib	60 mg	increased HDL concentrations; elevated CV risk and mortality rate	post hoc analysis of the Investigation of Lipid Level Management to Understand its Impact in Atherosclerotic Events (ILLUMINATE) trial	6661 diabetes mellitus patients	[24]
Anacetrapib	100 mg once daily	decreased coronary artery disease events associated with increased HDL concentration; decreased non-HDL cholesterol concentration	randomized, double-blind, placebo-controlled trial	30,449 adults with atherosclerotic vascular disease who were receiving intensive atorvastatin therapy	[25]
A1 Milano	5 weekly infusions of ETC-216 at 15 or 45 mg/kg	significant decrease in HDL cholesterol;strong cardioprotection	double-blind, randomized, placebo-controlled multicenter pilot trial	123 patients with ACS	[26]
CER-001	intravenous infusion of CER-001, 3 mg/kg	no decrease in coronary atherosclerosis on QCA or IVUS	randomized clinical trial	272 patients with an acute coronary syndrome	[27]
MDCO-216	2 h infusion of MDCO-216 (5–40 mg/kg)	no decrease in coronary atherosclerosis on QCA or IVUS	randomized, placebo-controlled, single ascending dose study	24 patients with documented CAD	[28]
